# Multiple systemic infections caused by *Rhodococcus equi*: a case report

**DOI:** 10.1099/acmi.0.000600.v4

**Published:** 2024-02-06

**Authors:** Ning Li, Changsheng Wu, Pengju Cao, Dongjie Chen, Falin Chen, Xiuqing shen

**Affiliations:** ^1^​ Shengli Clinical Medical College of Fujian Medical University, Fuzhou, PR China; ^2^​ Department of Clinical Laboratory, Fujian Provincial Hospital, Fuzhou, PR China; ^3^​ Fujian Medical University, Fuzhou, PR China

**Keywords:** multiple abscesses, non-human immunodeficiency virus, *Rhodococcus equi*, susceptibility

## Abstract

**Background.:**

*Rhodococcus equi* is one of the most important causes of zoonotic infections from grazing animals. It poses a particular risk to immunocompromised individuals, including those who are undergoing long-term immunosuppressive therapy.

**Case presentation.:**

We report a case of *Rhodococcus equi* infection in a 65-year-old man with a medical history of diabetes, hypertension, and Adult Still’s Disease, currently taking long-term hormone therapy. The non-human immunodeficiency virus (HIV)-infected patient had blood, lung tissue, and sputum samples infected with *Rhodococcus equi*. His condition initially failed to improve despite multiple therapies, including vancomycin and meropenem. Although his symptoms improved after shifting his antibiotics to cover for the causative agent, he did not completely recover upon hospital discharge.

**Conclusions.:**

In recent years, the number of *Rhodococcus equi* cases has increased. This report describes a lethal case of *Rhodococcus equi* infection in a patient without HIV.

## Data Summary

All data associated with this work is reported within the article.

## Background

*Rhodococcus equi* (*R. equi*), formerly *Corynebacterium equi*, is an infectious agent first isolated in 1923, and identified as a human pathogen in 1967 [1]. *R. equi* is an opportunistic pathogen that can infect any organ. However, immunocompromised (80 %) and immunocompetent individuals (30 %) have *R. equi* often present with pulmonary infections. More than 80 % of immunocompromised patients and approximately 30 % of immunocompetent patients present with bacteremia [2]. Currently, infection with *R. equi* rarely occurs, even among immunocompromised individuals [3]. The identification of *R. equi* in clinical samples should prompt clinicians to investigate the probable causes of the underlying immunocompromise. Though *R. equi* infections occur infrequently, its mortality rate can increase up to 50 % if the diagnosis is overlooked [4]. This report describes a case of *R. equi* infection in an immunocompromised patient.

## Case presentation

On 6 May 2020, a 65-year-old male was admitted due to a 20 day history of weakness and numbness of the right upper extremity. This extremity weakness worsened in the last 4 days. He has a past medical history of diabetes mellitus, hypertension, and Adult Still’s Disease. His Still’s Disease is currently being managed with methylprednisolone.

On physical examination, his blood pressure was 129/77 mmHg, pulse rate was 88 min^−1^, and body temperature was 36.7°C. An increase in muscle tone was observed in both upper extremities. His muscle strength was 5/5 in the left upper extremity, and 4/5 in the right upper extremity. Additionally, his biceps tendon reflex, triceps tendon reflex, radial membrane reflex (+++), knee tendon reflex (++++), and Achilles tendon reflex (++) were heightened.

On hospital day 1, he developed a fever (38.7°C), prompting a blood examination. On hospital day 2, repeat blood cultures were collected. The first set of blood cultures tested positive after 2 days. However, subsequent blood culture tests conducted 1 week later returned negative. Cranial computed tomography (CT) revealed multiple abscesses in the right frontal and occipital lobes, and both parietal lobes. On auscultation, he had coarse and minimal breath sounds. Moreover, chest CT revealed necrosis and abscesses in the lower lobes of the left lung. Tenderness in the right subabdominal McCormack point was also observed. An abdominal CT revealed an abscess in the left kidney. Laboratory examination revealed an elevated white blood cell (WBC) count (12300 /µl), neutrophils 86.4 %, decreased haemoglobin (120 g l^−1^), normal platelet count (274000 /µl), and increased C-reactive protein (84.9 mg l^−1^). Immunological testing revealed the following results: CD4 lymphocytes 40 %, CD8 lymphocytes 35 %, and CD4–CD8 ratio of 1.14. Humoral immunity testing revealed the following results: immunoglobulin (IgG of 6.09 g l^−1^, IgA of 0.92 g l^−1^, IgM of 0.94 g l^−1^, complement C3 of 0.854 g l^−1^, and complement C4 of 0.081 g l^−1^). He was negative in an HIV screening exam. But his HBsAg, HBeAb, and HBcAb were positive.

On 8 May 2020, he was started on meropenem and vancomycin. On 14 May 2020, *R. equi* was isolated from his blood culture. Antimicrobial susceptibility testing was not performed because it was a rare bacterium that lacked a standardized drug-sensitive breakpoint. Though he was given broad-spectrum antibiotics, his symptoms failed to improve even after receiving intravenous vancomycin and meropenem for 20 days.

Antimicrobial susceptibility testing with broth microdilution was performed on 11 June 2020. The cultured organism was found to be resistant *in vitro* to penicillin, erythromycin, and vancomycin, and susceptible to ciprofloxacin, levofloxacin, imipenem, linezolid, and compound sulfamethoxazole (Table 1) according to CLSI M45 A3. Drug-sensitive breakpoints were referred to *Staphylococcus aureus*. His treatment regimen was then altered to linezolid and meropenem. However, these medications were discontinued due to drug-induced bone marrow suppression and liver injury. The patient later suffered liver failure and died at home.

**Table 1.** Antimicrobial susceptibility of *Rhodococcus equi*

Antimicrobials	MIC (mg l^−1^)
Imipenem	0.25
Erythromycin	32
Penicillin	4
Ciprofloxacin	0.5
Levofloxacin	1
Gentamicin	0.5
Vancomycin	256
Linezolid	2

MIC, minimum inhibitory concentration.

**Fig. 1.**
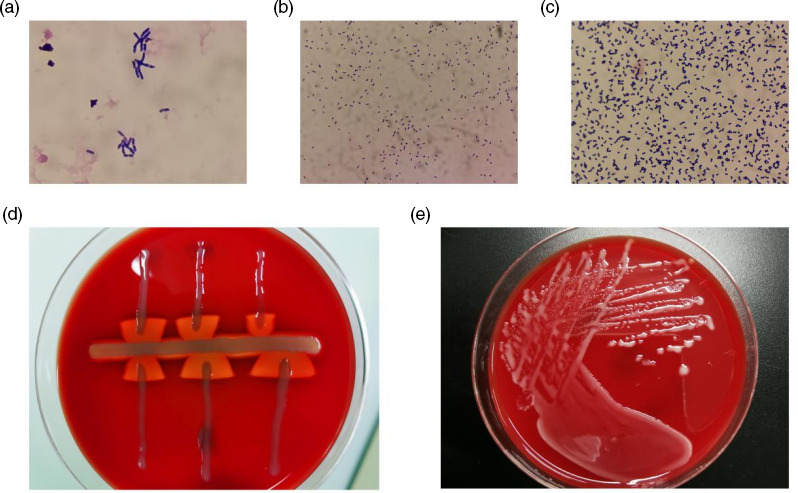
(a): Gram-stained blood culture yielded Gram-positive bacillus; (b): Pleomorphic Gram-positive coccobacilli was observed after 24 h of incubation; (c): Gram-positive coccobacilli was observed after 72 h of incubation. (d): The Christie–Atkins–Munch–Peterson test; (e): Colony morphology after 48 h of pure culture.

*R. equi* is a non-motile Gram-positive obligate aerobic coccobacillus (Fig. 1a–c). The Christie–Atkins–Munch–Peterson test was performed on blood agar plates with *Staphylococcus aureus* (ATCC 25923). After 24 h of incubation, *R. equi* hemolysed in the form of an arrowhead near *S. aureus* (Fig. 1d). After 48 h of incubation, aerobic non-hemolytic, mucoid, and white colonies were evident (Fig. 1e). The organism was identified as *R. equi* by MALDI-TOF VITEK MS. Conventional methods revealed catalase positivity, urease positivity, oxidase negativity, gelatinase negativity, and a failure to oxidize or ferment carbohydrates.

## Discussion

This report presents a case of *R. equi* infection in a patient without HIV infection. The patient had an autoimmune disease that required hormone therapy, which resulted in a compromised cellular and humoral immunity. *R. equi* belongs to the genus Rhodococcus and family Nocardiaceae [5]. Initially, in 1983, only 12 cases of human *R. equi* infections had been reported [6]. However, within the subsequent 15 years, the number of reported cases increased significantly, reaching at least 100 cases by 1988 [7–14]. This phenomenon coincides with the era of increased HIV infection and advances in organ transplantation and cancer treatment [15]. It may also be attributed to the improvements in the laboratory techniques used to isolated *R. equi*.

Due to its similarity to tuberculosis, Nocardia, and some Corynebacteria, laboratories tend to misidentify Rhodococcus as one of these bacteria [16]. Diagnosis is often delayed because Rhodococcus can masquerade as other infections, leading to incorrect treatment plans and delayed treatment. Therefore, appropriate clinical suspicion and good coordination and communication between clinical and laboratory personnel are necessary. Gram-staining and colony patterns of *R. equi*, according to the culture stage, should be promptly observed to diagnose this bacteria.

*R. equi* infection is often associated with HIV, and most individuals infected with *R. equi* exhibit some form of immunocompromise [17]. However, *R. equi* infections among immunocompetent patients are rare. The isolation of Gram-positive rods from immunosuppressed patients should lead to the suspicion of an *R. equi* infection. Immunocompromised patients often present with pulmonary involvement, with necrotizing pneumonia as the most frequent presentation [17]. *R. equi* infection can be life-threatening, and the required treatment is often extensive. The clinical manifestations of *R. equi* infection vary diversly; and approximately 80 % present with pulmonary involvement [15]. The patient in this case presented with neurological symptoms. Blood, lung tissue, and sputum samples were infected with *R. equi*.

Hematogenous spread is relatively common, mostly owing to its dissemination from the lungs [16]. The patient’s *R. equi* infection was considered to have originated from the respiratory tract. Since his blood culture was positive on hospital Day 1, but was negative on Day 2, it is necessary to collect the samples in a timely manner, before the initiation of antibiotics.

*R. equi* is found in the soil and horse faeces, and has sporadically been reported to cause infection in other domestic animals, such as cattle, pigs, sheep, and goats [15, 18–20]. However, no environmental exposure that might have predisposed the patient to the *R. equi* infection was noted. Owing to the rarity of *R. equi* infection, a standard treatment regimen for this disease has not been established; however, a combination of antibiotics has been recommended [21]. The Sanford Guide to Antimicrobial Therapy recommends that the first line of anti-infective treatment of *R. equi* is azithromycin, levofloxacin, rifampicin, or a combination of the two. Second-line drugs would include vancomycin, imipenem, levofloxacin, azithromycin, or rifampicin [22]. Penicillin, cephalosporins, clindamycin, tetracycline, and cotrimoxazole should be avoided. Since the patient had a poor response to vancomycin and meropenem, his drug regimen was altered based on the results of his antibiotic susceptibility testing. Reports have shown that *R. equi* may be susceptible to vancomycin *in vitro*. However, *R. equi* is a facultative intracellular parasite that cannot achieve its desired intracellular concentration, resulting in reduced activity against intracellular *R. equi* and treatment failure [23]. The patient’s symptoms began to improve after adjusting his antibiotic therapy. However, the patient did not fully recover upon discharge. This highlights the cruciality of timely susceptibility testing for *R. equi* infections.

This study reports a case of an *R. equi* infection in a patient without HIV infection. *R. equi* was isolated in blood, lung tissue, and sputum samples. Unfortunately, he was unresponsive to the initial antibiotics given to him. Accurate and timely feedback from laboratory personnel to clinicians will enable patients to receive appropriate treatment plans. This case elicits the pathogenic potential of *R. equi*, even in patients without HIV. Moreover, it highlights the value of antibiotic susceptibility testing. Furthermore, this case underscores the presence of *R. equi* in a patient without HIV, but was still immunocompromised due to the intake of medications for Still’s disease. Therefore, clinicians should be vigilant of probable infections caused by *R. equi*.
